# Electrochemical and spectroscopic properties of twisted dibenzo[*g*,*p*]chrysene derivatives

**DOI:** 10.3762/bjoc.18.96

**Published:** 2022-08-03

**Authors:** Tomoya Imai, Ryuhei Akasaka, Naruhiro Yoshida, Toru Amaya, Tetsuo Iwasawa

**Affiliations:** 1 Department of Information and Basic Science, Graduate School of Science, Nagoya City University, 1, Yamanohata, Mizuho-cho, Mizuho-ku, Nagoya, Aichi 467-8501, Japan; 2 Department of Materials Chemistry, Ryukoku University, Seta, Otsu, Shiga, 520-2194, Japan

**Keywords:** DFT calculation, dibenzo[*g*,*p*]chrysenes, fluorescent compounds, oxidation, polycyclic aromatic hydrocarbon (PAH), twisted acenes

## Abstract

Dibenzo[*g*,*p*]chrysene (DBC), which consists of a twisted naphthalene core with four fused benzene rings, is a promising framework for organic electronic materials. Therefore, the research for structure–property relationships is important for the design of DBC-based materials. Here, the electrochemical and spectroscopic properties of DBC derivatives were investigated, and the effects of substituents and torsion of the naphthalene moiety were examined based on density functional theory (DFT) calculations. All the substituted DBC derivatives showed higher oxidation potentials than that for **DBC-H**, even for compounds that contained an electron-donating group such as **DBC-Me** and **DBC-SMe**. DFT calculations clearly indicate that these higher oxidation potentials are due to the ineffective conjugation of the MeO group, which is oriented perpendicular to the benzene ring because of the steric repulsion of substituents on both sides. More specifically, the inductive effect of the MeO group is dominant rather than the mesomeric effect when the substituent is located at both sides of the MeO group. Concerning the torsion of the naphthalene moiety, the twisting results in a slight increase in the HOMO and a slight lowering of the LUMO. The twisting effect is much smaller than the conjugation effect of the MeO group. Absorption spectra of all the substituted DBC derivatives also showed a red-shift as compared to that for **DBC-H**. Concerning the luminescence, a strong photoluminescence was observed for **DBC-H** and **DBC-Si**.

## Introduction

**Figure 1:**
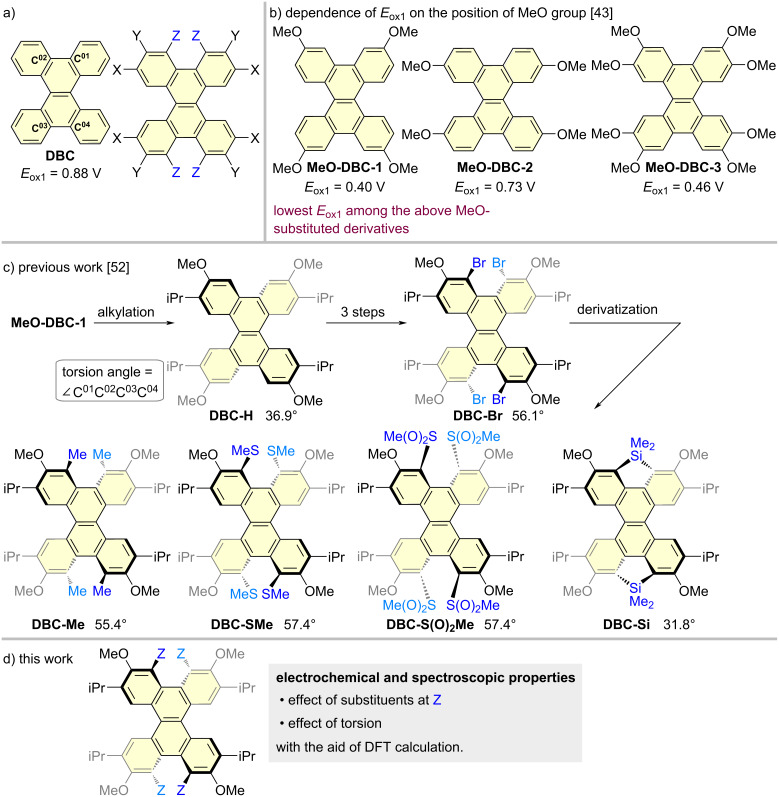
a) DBC. b) Dependence of *E*_ox1_ on the position of the MeO groups [43]. c) Previous work [52]. d) This work.

Polycyclic aromatic hydrocarbons (PAHs) have attracted interest as potential electronic and optoelectronic materials [1–12]. Non-planar PAHs have been extensively investigated from the viewpoint of their synthetic challenge and/or for the development of functional organic materials [13–22]. Among such PAHs, twisted acenes are an interesting class of compounds due to their characteristic structures and conjugation systems [23–25]. Dibenzo[*g*,*p*]chrysene (DBC), which consists of a twisted naphthalene core with four fused benzene rings (Figure 1a) [26], is a promising framework for serving as organic semiconductors, dyes, liquid crystals, and light-emitting materials. A number of substituted DBCs have been reported in this context [27–46]. To develop charge-transport materials, Rathore et al. reported on the stability of radical cations of DBCs with MeO groups located at X and/or Y (**MeO-DBC-1**, **MeO-DBC-2**, and **MeO-DBC-3**, Figure 1b) [43]. The first oxidation potential (*E*_ox1_) of **MeO-DBC-1** was reported to be 0.40 V (based on Fc/Fc^+^), which is 0.48 V lower than that of **DBC**. In contrast, when a MeO group is introduced at the X position (**MeO-DBC-2**), the *E*_ox1_ is lower by only 0.15 V than that of **DBC**. It has also been reported that the oxidation potential of **MeO-DBC-3**, in which the MeO groups are attached at both X and Y, is 0.06 V higher than that for **MeO-DBC-1**. These remarkable substituent effects are an interesting and important finding for molecular design, but the effects of X and Z substituents and the twisting of the naphthalene moiety have not been reported.

We previously studied the synthesis of solution-processable DBC derivatives with various substituents attached [47–51]. We also recently reported on a synthetic strategy for preparing DBC derivatives using **DBC-H** with four isopropyl groups at X as a key template for the derivatization (Figure 1c). Based on this strategy, various substituents were introduced at Z to produce **DBC-Br**, **DBC-Me**, **DBC-SMe**, **DBC-S(O)_2_Me**, and **DBC-Si** (Figure 1c) [52]. The structures of all these derivatives were determined by X-ray crystallographic analysis, in which torsion angles were varied in a range from 31.8º (**DBC-Si**) to 57.4º (**DBC-S(O)_2_Me**) [52]. These DBC derivatives have four methoxy moieties at the Y position, which aroused our interest concerning the stability of those oxidation states.

Herein, we report on the electrochemical and spectroscopic properties of the DBC derivatives, where the effects of substituents and torsion were examined with the aid of DFT calculations. Consequently, the findings revealed that the substitution at the Z position induces a change in the conformation of the MeO groups, making the conjugation of the MeO groups ineffective, thus resulting in the lowering of both HOMO and LUMO energy levels. Concerning the twisting, the effect to the HOMO and LUMO energy levels was found to be small. We anticipate that the impact of diverse substituents and torsion angles on the chemical properties would be beneficial in terms of creating DBC-based materials.

## Results and Discussion

### Electrochemical properties

**Figure 2:**
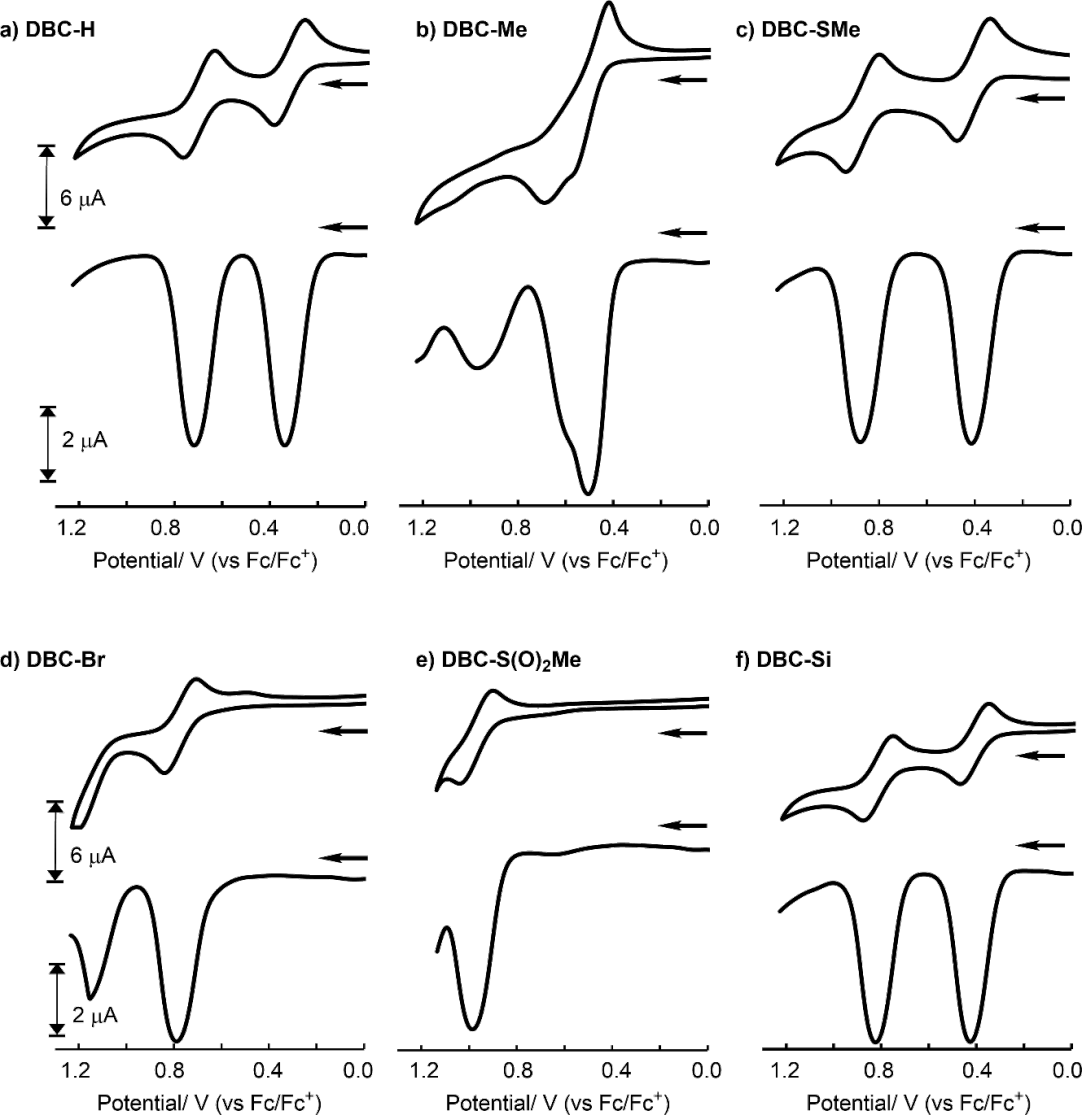
CVs and SWVs of DBC derivatives in CH_2_Cl_2_ (≈1.0 × 10^−3^ M, see Supporting Information File 1 for details) including 5.0 × 10^−2^ M NBu_4_BF_4_ as a supporting electrolyte under Ar at 298 K (working electrode: Pt, scan rate: 100 mV/s and 40 mV/s for CV and SWV measurements, respectively).

Cyclic voltammograms (CVs) and square-wave voltammograms (SWVs) were measured for **DBC-H**, **DBC-Me**, **DBC-SMe**, **DBC-Br**, **DBC-S(O)_2_Me**, and **DBC-Si** (Figure 2) [53]. Table 1 summarizes the first and second oxidation potentials based on Fc/Fc^+^ (*E*_ox1_ and *E*_ox2_) determined from the SWVs, together with the torsion angles determined from the X-ray crystal structures [52], the HOMO and LUMO levels determined from DFT calculations [52,54] and estimated based on *E*_ox1_. The voltammogram of **DBC-H** exhibited a reversible, two-step, two-electron redox process, with *E*_ox1_ and *E*_ox2_ values of 0.34 V and 0.72 V, respectively (Figure 2a). The value of *E*_ox1_ is 0.06 V lower than that of **MeO-DBC-1** which does not contain isopropyl groups. This is in contrast to **MeO-DBC-3**, in which four MeO groups are introduced in place of the isopropyl groups, which has a 0.06 V higher oxidation potential than that of **MeO-DBC-1**. This indicates that alkyl substituents in the X position are effective in stabilizing the radical cation, thus making it more susceptible to oxidation. Unlike **DBC-H**, an irreversible voltammogram was observed in case of **DBC-Me** (Figure 2b). The first oxidation potential obtained from the SWV was 0.51 V, which is 0.17 V higher than that of **DBC-H**. This higher oxidation potential is somewhat surprising, which is discussed in the next paragraph based on DFT calculations. The CV of **DBC-SMe** showed a reversible two-electron redox process, with *E*_ox1_ and *E*_ox2_ values of 0.41 V and 0.88 V, respectively (Figure 2c) [53]. It is interesting to note that **DBC-SMe** exhibited a higher oxidation potential than **DBC-H** despite the electron-donating nature due to mesomeric effects based on lone pairs of sulfur atoms. In the CV of **DBC-Br**, a one-electron redox was observed as a reversible process, but a second redox process was not observed (Figure 2d). On the other hand, both the first and second oxidation processes were observed in the SWV of **DBC-Br** (*E*_ox1_ and *E*_ox2_ are 0.79 V and 1.15 V, respectively). **DBC-S(O)_2_Me** with the electron-withdrawing substituents resulted in a reversible oxidation wave, but only a one-electron redox process could be observed due to the limitations of the solvent (Figure 2e). The potential of 0.98 V is the highest among the compounds measured in this study. To investigate the reduction behaviour, **DBC-S(O)_2_Me** was measured in the low potential region. A peak, which appeared to be the one-electron reduction peak, was observed at −2.25 V (see Figure S1 in Supporting Information File 1). Finally, the CV of the silole-fused **DBC-Si** was investigated and the results indicated a reversible two-electron redox process (*E*_ox1_ and *E*_ox2_ are 0.43 V and 0.82 V, respectively, Figure 2f). These values for *E*_ox1_ and *E*_ox2_ for **DBC-Si** are slightly higher than those of **DBC-H**. The obtained electrochemical data were nearly consistent with the trend of the values for HOMO obtained based on DFT calculations.

**Table 1:** Electrochemical data, torsion angles determined from the X-ray crystal structures, and HOMO and LUMO levels for DBC derivatives^a^.

compounds	*E*_ox1_ [V]^b^	*E*_ox2_ [V]^b^	torsion angle [°]^c^	HOMO [eV]^d^ (the estimated values based on experimental data in parentheses)^e^	LUMO [eV]^d^

**DBC-H**	0.34	0.72	36.9	−4.64 (−5.4)	−0.87
**DBC-Me**	0.51	0.96	55.4	−4.81 (−5.6)	−1.22
**DBC-SMe**	0.41	0.88	57.4	−5.00 (−5.5)	−1.42
**DBC-Br**	0.79	1.15	56.1	−5.24 (−5.9)	−1.71
**DBC-S(O)** **_2_** **Me**	0.98	–	57.4	−5.56 (−6.1)	−2.00
**DBC-Si**	0.43	0.82	31.8	−4.80 (−5.5)	−1.09

^a^Concentration: Around 1.0 × 10^−3^ M in CH_2_Cl_2_ (for detailed values, see Supporting Information File 1) containing 5.0 × 10^−2^ M NBu_4_BF_4_ as a supporting electrolyte. SWVs were recorded at a platinum electrode at 298 K under Ar. ^b^Based on Fc/Fc^+^. ^c^The values obtained from X-ray crystallographic analyses [52]. ^d^The values obtained from DFT calculations at B3LYP6-31G(d,p) [52,54]. ^e^The energy values of HOMO were estimated based on the following equation *E*_HOMO_ = −(*E*_ox1 vs Fc+/Fc_ + 5.1) [55].

### Theoretical calculations

**Figure 3:**
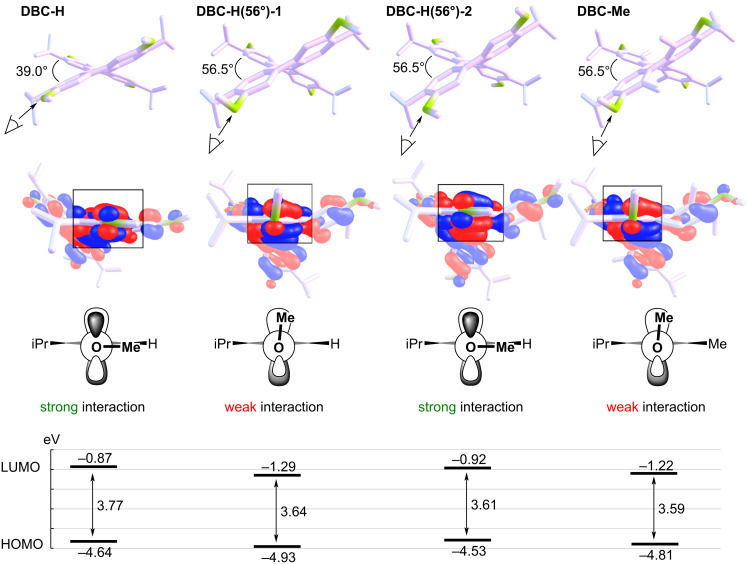
DFT-optimized structures, orbital drawings of HOMO, schematic drawings of orbital interaction, and energy diagrams for **DBC-H**, **DBC-H(56°)-1**, **DBC-H(56°)-2**, and **DBC-Me**.

DFT calculations were performed to clarify the reasons for the oxidation potentials [52,54,56]. To investigate the effects of the torsion of the naphthalene moiety and the conformation of the MeO group on the oxidation potential of these materials, hypothetical compounds **DBC-H(56°)-1** and **DBC-H(56°)-2** were created, respectively. In **DBC-H(56°)-1**, the atoms are fixed except for the Me group of **DBC-Me** (torsion angle = 56.5°), and the Me group is changed to H. **DBC-H(56°)-2** is the same structure as **DBC-H(56°)-1** except for the MeO group conformation. Optimizations of **DBC-H(56°)-1** and **DBC-H(56°)-2** based on DFT calculations were performed by fixing the atoms, as described above [56]. The conformations of the MeO group in **DBC-H(56°)-1** and **DBC-H(56°)-2** are nearly perpendicular (98.3°) to and parallel (179.8°) to the benzene ring, respectively (Figure 3 and Table 2). The results were compared to those for **DBC-H** and **DBC-Me** (Figure 3). To examine the torsional effect, **DBC-H** (torsion angle = 39.0°) and **DBC-H(56°)-2** (torsion angle = 56.5°) were compared. The HOMO level of the highly twisted **DBC-H(56°)-2** was 0.09 eV higher than that of the less twisted **DBC-H**. Conversely, the LUMO level of the highly twisted **DBC-H(56°)-2** was 0.05 eV lower than the less twisted **DBC-H**. As a result, the HOMO–LUMO gap of **DBC-H(56°)-2** becomes smaller than that of **DBC-H**. This is consistent with the trend reported for twisted acenes [57]. The conformational effect of the MeO group was investigated by comparison of **DBC-H(56°)-1** (perpendicular to the benzene ring, 98.3°) with **DBC-H(56°)-2** (parallel to the benzene ring, 179.8°). Consequently, both the HOMO and LUMO levels of **DBC-H(56°)-2** are higher than those of **DBC-H(56°)-1** by −0.40 eV and −0.37 eV, respectively. This is likely attributed by the effect of conjugation for the orbital of an oxygen atom as shown in the schematic drawing in Figure 3. When the conformation of the MeO group is almost parallel to the benzene ring, the strong orbital interaction between the orbitals on the oxygen and adjacent carbon atoms is possible in HOMO (the orbital drawings are also shown in Figure S2 in Supporting Information File 1). In this case, the mesomeric effect of an oxygen atom is dominant. On the other hand, when the conformation of the MeO group is almost perpendicular to the benzene ring, the interaction between orbitals on the oxygen and adjacent carbon atoms becomes weak in the case of HOMO. In this case, the inductive effect of an oxygen atom can be dominant. Thus, the substituents at the Z position allow the MeO group to be oriented perpendicular to the benzene ring, which results in the lowering of both the HOMO and LUMO (Figure 3). In **DBC-Me**, the lowering of the HOMO based on the inductive effect offsets the increase in HOMO due to the electron-donating nature of the Me group. This can account for the observed higher *E*_ox1_ for **DBC-Me** than that for **DBC-H**.

**Table 2:** Dihedral angles for the DFT-optimized structures of DBC derivatives [B3LYP6-31G(d,p)].

compounds	substituent Z	dihedral angle of ABCD [°]	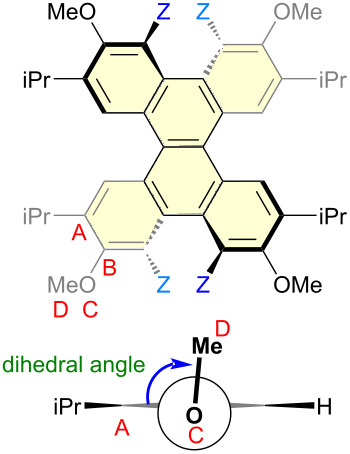

**DBC-H**	H	179.6	
**DBC-H(56°)-1**	H	98.3	
**DBC-H(56°)-2**	H	179.8	
**DBC-Me**	Me	98.3	
**DBC-SMe**	SMe	105.7	
**DBC-Br**	Br	111.1	
**DBC-S(O)** **_2_** **Me**	S(O)_2_Me	97.8	
**DBC-Si**	-SiMe_2_-	156.6	

Other derivatives were also examined. The dihedral angles are summarized in Table 2. The MeS group is an electron-donating group and may increase the HOMO, but the HOMO level of **DBC-SMe** is lower than that of **DBC-H**, as shown in the electrochemical study and by DFT calculations (Table 1). This is considered to be due to the contribution of the inductive effect of the MeO group by ineffective conjugation. In **DBC-Br** and **DBC-S(O)_2_Me**, both the HOMO and LUMO are lower, which can be attributed to the combined effects of their electron-withdrawing by Br and S(O)_2_Me groups and ineffective conjugation of the MeO group. In the case of **DBC-Si**, where the dihedral angle of the MeO group is 156.6°, both the HOMO and LUMO are lower than those for **DBC-H**. Although it is not perpendicular, the lower energy levels for HOMO and LUMO can be accounted for by the ineffective conjugation of the MeO group.

### Spectroscopic properties

**Figure 4:**
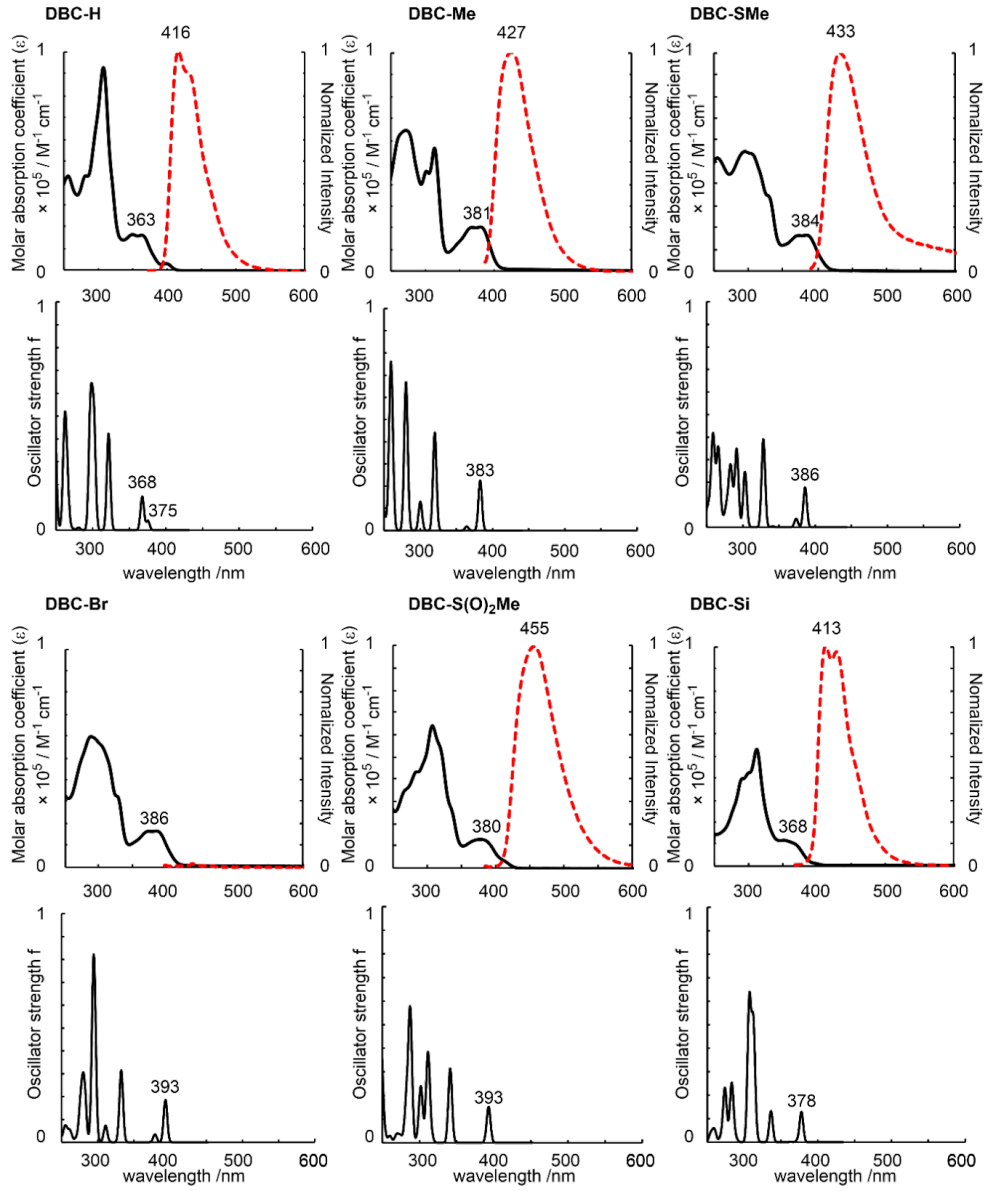
Absorption (solid line) and photoluminescence (dotted red line) spectra (upper graphs) in CH_2_Cl_2_ and simulations of absorption based on TD-DFT calculations [lower graphs, TD-B3LYP-D3/6-31G(d,p)//B3LYP/6-31G(d,p)] for DBC derivatives.

Absorption and photoluminescence spectra and the simulations of absorption based on TD-DFT calculations [58] are shown in Figure 4 (see Figure S3 in Supporting Information File 1 for excited spectra). The spectral data are summarized in Table 3. The TD-DFT calculations reproduce the absorption spectra quite well. The longest absorption peak is attributed to the transition from HOMO to LUMO and HOMO-1 to LUMO+1 (see Tables S1–S6 in Supporting Information File 1). The trend for the order of optical band gap is roughly consistent with that of the HOMO–LUMO gap obtained from DFT calculation [52].

**Table 3:** Absorption and photoluminescence spectral data of DBC derivatives in CH_2_Cl_2_.

compounds	absorption λ_max_ [nm] molar absorption coefficient ε [M^−1^·cm^−1^] in parentheses	optical band gap^a^ [eV]	photoluminescence λ_max_ [nm]	quantum yield [%]^b^

**DBC-H**	363 (16200)	2.95	416	28
**DBC-Me**	381 (20300)	2.91	427	21
**DBC-SMe**	384 (16500)	2.88	433	3
**DBC-Br**	386 (16100)	2.86	–^c^	–^c^
**DBC-S(O)** **_2_** **Me**	380 (12900)	2.82	455	6
**DBC-Si**	368 (9500)	2.97	413	11

^a^Estimated from the absorption edge. ^b^Measured based on the absolute quantum yield method using an integrating sphere. ^c^Too weak photoluminescence to measure.

In the photoluminescence spectra, the luminescence of **DBC-Br** was very weak. On the other hand, **DBC-H**, **DBC-Me**, and **DBC-Si** showed relatively strong photoluminescences with quantum yields of 28%, 21%, and 11%, respectively (Table 3). The photoluminescence wavelengths were shifted toward longer wavelengths in the order of **DBC-Si**, **DBC-H**, **DBC-Me**, **DBC-SMe**, and **DBC-S(O)_2_Me**. Of these, the Stokes shift for **DBC-S(O)_2_Me** was the largest, which is due to the electron-withdrawing nature of the S(O)_2_Me group.

## Conclusion

The electrochemical and spectroscopic properties of DBC derivatives were investigated, and the effects of substituents and torsion of the naphthalene moiety were discussed based on DFT calculations. It was also found that introducing a substituent at Z position resulted in a higher oxidation potential than that for **DBC-H**, even for compounds that contained electron-donating groups, such as **DBC-Me** and **DBC-SMe**. DFT calculations clearly indicate that this is due to the ineffective conjugation of the MeO group which is oriented perpendicular to the aromatic ring because of the steric repulsion of substituents on both sides. More specifically, the inductive effect of the MeO group is dominant rather than the mesomeric effect when the substituent is present at the Z position. Concerning the torsion of the naphthalene moiety, the twisting caused a slight increase in the HOMO and a slight lowering of the LUMO. The twisting effect is much smaller than the conjugation effect of the MeO group. Absorption spectra of all the substituted DBC derivatives also showed a red-shift as compared to that for **DBC-H**. Concerning photoluminescence, a strong photoluminescence was observed for **DBC-H** and **DBC-Si**. The findings reported in this study will be useful for the molecular design of such materials, and could lead to electronic material applications in the future.

## Supporting Information

File 1 Figures S1–S3, Tables S1–S6, general, experimental procedure, and cartesian coordinates of optimized structures obtained based on the theoretical calculation.
