# Supplementary material for: Electrochemical and spectroscopic properties of twisted dibenzo[g,p]chrysene derivatives
Source: Beilstein J Org Chem. 2022 Aug 3;18:963–71. doi: 10.3762/bjoc.18.96 (PMC9359188; doi:10.3762/bjoc.18.96)
Supplement: File 1 — Figures S1–S3, Tables S1–S6, general, experimental procedure, and cartesian coordinates of optimized structures obtained based on the theoretical calculation. [file Beilstein_J_Org_Chem-18-963-s001.pdf]

BEILSTEIN JOURNAL OF ORGANIC CHEMISTRY

Supporting Information

for

Electrochemical and spectroscopic properties of twisted dibenzo[*g*,*p*]chrysene derivatives

Tomoya Imai, Ryuhei Akasaka, Naruhiro Yoshida, Toru Amaya and Tetsuo Iwasawa

Beilstein J. Org. Chem. **2022,** *18,* 963–971. doi:10.3762/bjoc.18.96

Figures S1–S3, Tables S1–S6, general, experimental procedure, and cartesian coordinates of optimized structures obtained based on the theoretical calculation

License and Terms: This is a supporting information file under the terms of the Creative Commons Attribution License (https://creativecommons.org/licenses/by/4.0). Please note that the reuse, redistribution and reproduction in particular requires that the author(s) and source are credited and that individual graphics may be subject to special legal provisions.
The license is subject to the *Beilstein Journal of Organic Chemistry* terms and conditions: (https://www.beilstein-journals.org/bjoc/terms)

Table of contents

1. **Figures (S1-S3)** **S3**
2. **Tables (S1-S6)** **S5**
3. **General** **S7**
4. **Experimental Procedure** **S8**
5. **Cartesian Coordinates of Optimized Structures** **S8**

1. Figures (S1-S3)

DBC-SMe

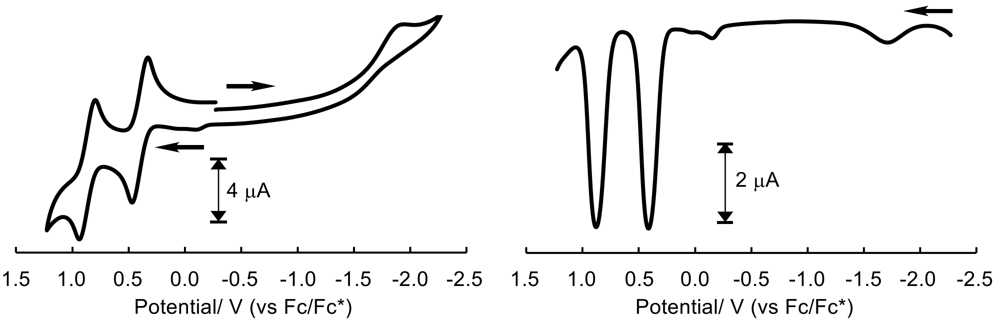

DBC-S(O)$_2$Me

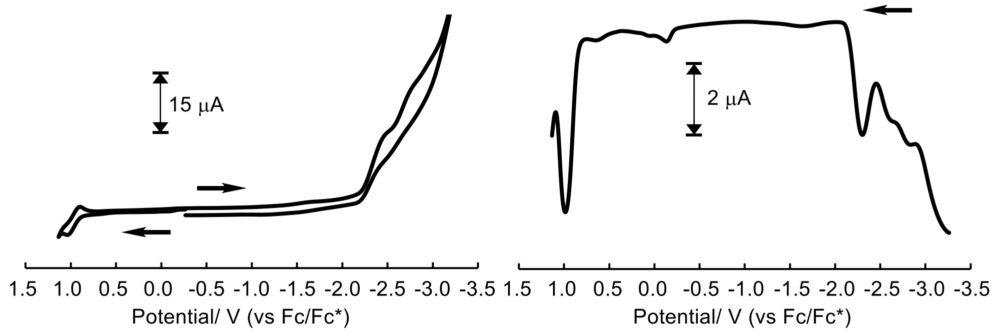

Figure S1. CVs and SWVs of **DBC-SMe** and **DBC-S(O)$_2$Me** in CH_2Cl_2 (~1.0 x 10^{-3} M, see 4. Experimental Procedure for detail) including 5.0 x 10^{-2} M NBu_4BF_4 as a supporting electrolyte under Ar at 298 K (working electrode: Pt, scan rate: 100 mV/s and 40 mV/s for CV and SWV measurements, respectively).

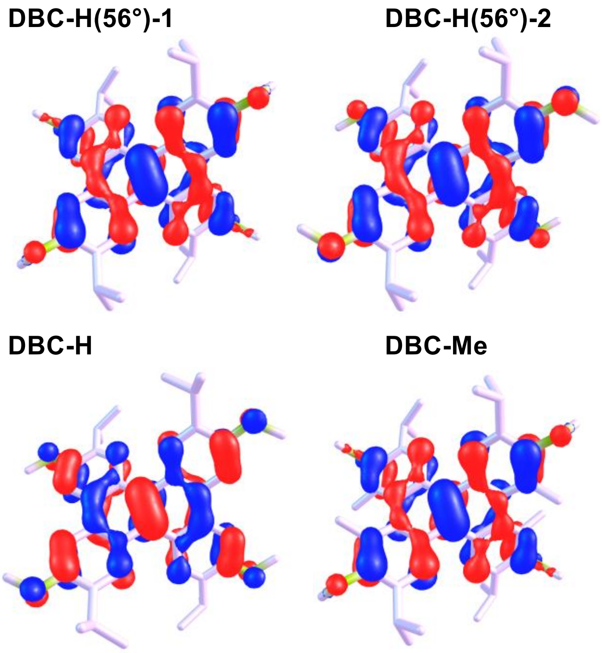

Figure S2. Orbital drawings of HOMO for DBC derivatives.

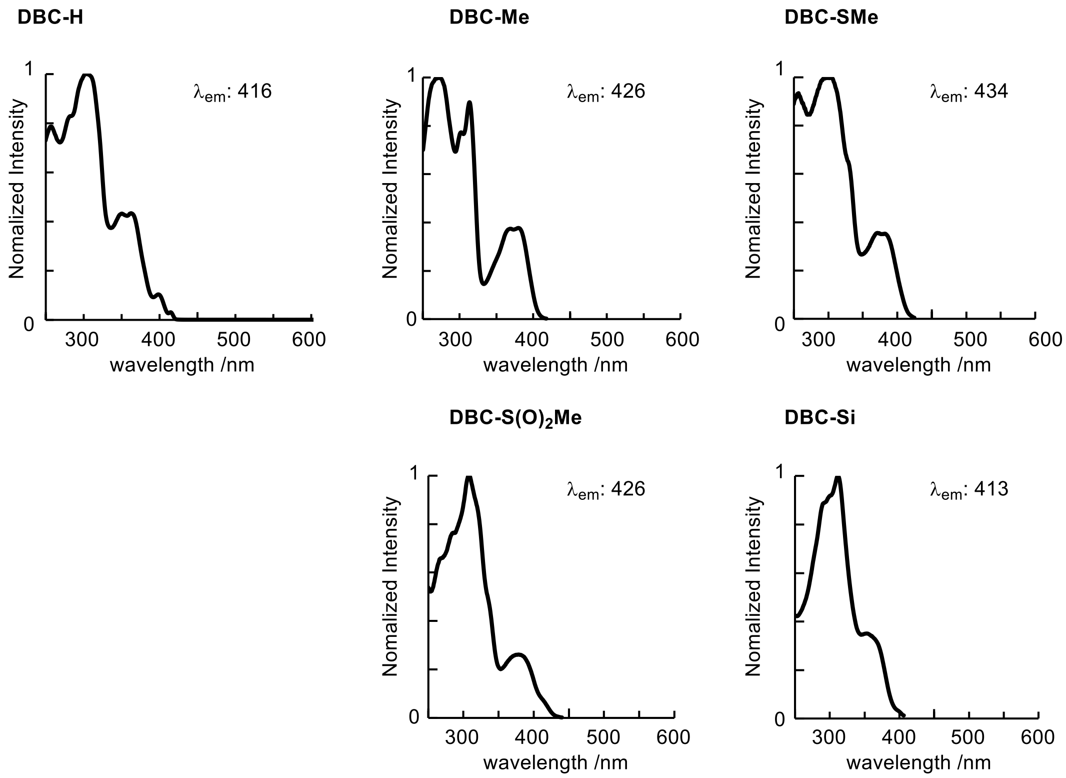

Figure S3. Excited spectra for DBC derivatives in CH_2Cl_2.

Table S1. Wavelengths and oscillator strengths (*f*) for **DBC-H** estimated by TD-DFT calculation [TD-B3LYP-D3/6-31G(d,p)// B3LYP/6-31G(d,p)]

Excited state	Wavelength (nm)	*f*	Transition ($\psi_{xxx} \rightarrow \psi_{yyy}$)					
1	375.36	0.0409	165 → 167	-0.33401	166 → 168	0.61777		
2	367.64	0.1474	165 → 168	0.22931	166 → 167 (HOMO→LUMO)	0.66346		
3	348.78	0.0000	164 → 167	-0.18876	166 → 169	0.67616		
4	321.69	0.4225	165 → 167	0.57466	166 → 168	0.31408	166 → 170	-0.24859
5	301.66	0.4664	164 → 169	-0.11143	165 → 168	0.65429	166 → 167 (HOMO→LUMO)	-0.21024

Table S2. Wavelengths and oscillator strengths (*f*) for **DBC-Me** estimated by TD-DFT calculation [TD-B3LYP-D3/6-31G(d,p)// B3LYP/6-31G(d,p)]

Excited state	Wavelength (nm)	*f*	Transition ($\psi_{xxx} \rightarrow \psi_{yyy}$)					
1	382.98	0.2250	181 → 184	0.14522	182 →183 (HOMO→LUMO)	0.68676		
2	364.35	0.0181	181 → 183	-0.40652	182 → 184	0.57291		
3	344.69	0.0000	180 → 183	-0.32512	182 → 185	0.61825		
4	320.20	0.4413	181 → 183	0.55590	182 → 184	0.39632	182 → 186	-0.14875
5	307.82	0.0000	180 → 183	0.61328	182 → 185	0.32188		

Table S3. Wavelengths and oscillator strengths (*f*) for **DBC-SMe** estimated by TD-DFT calculation [TD-B3LYP-D3/6-31G(d,p)// B3LYP/6-31G(d,p)]

Excited state	Wavelength (nm)	*f*	Transition ($\psi_{xxx} \rightarrow \psi_{yyy}$)					
1	386.11	0.1776	212 → 216	0.15435	214 → 215 (HOMO→LUMO)	0.68395		
2	373.77	0.0382	212 → 215	-0.33988	214 → 216	0.61244		
3	354.45	0.0000	208 → 215	-0.21817	210 → 215	-0.12908	214 → 217	0.64660
4	341.43	0.0041	213 → 215	0.69332				
5	328.47	0.3898	211 → 216	0.10352	212 → 215	0.58018	214 → 216	0.32907
					214 → 218	0.18299		

Table S4. Wavelengths and oscillator strengths (*f*) for **DBC-Br** estimated by TD-DFT calculation [TD-B3LYP-D3/6-31G(d,p)// B3LYP/6-31G(d,p)]

Excited state	Wavelength (nm)	*f*	Transition ($\psi_{xxx} \rightarrow \psi_{yyy}$)					
1	393.50	0.1852	233 → 236	0.14200	234 → 235 (HOMO→LUMO)	0.68627		
2	378.99	0.0344	233 → 235	-0.33512	234 → 236	0.61613		
3	362.28	0.0000	232 → 235	0.27025	234 → 237	0.64431		
4	332.41	0.3147	233 → 235	0.58566	234 → 236	0.32549	234 → 238	0.20022
5	320.89	0.0000	232 → 235	0.63722	234 → 237	-0.27031		

Table S5. Wavelengths and oscillator strengths (*f*) for **DBC-S(O)$_2$Me** estimated by TD-DFT calculation [TD-B3LYP-D3/6-31G(d,p)// B3LYP/6-31G(d,p)]

Excited state	Wavelength (nm)	*f*	Transition ($\psi_{xxx} \rightarrow \psi_{yyy}$)					
1	393.30	0.1311	245 → 248	0.18016	246 → 247 (HOMO→LUMO)	0.67649		
2	391.34	0.0269	245 → 247	-0.30004	246 → 248	0.63429		
3	375.53	0.0004	244 → 247	0.19897	246 → 249	0.67199		
4	341.34	0.3149	245 → 247	0.60776	246 → 248	0.29604	246 → 251	0.18560
5	318.88	0.0003	242 → 248	-0.11521	244 → 247	0.65664	246 → 249	-0.19851

Table S6. Wavelengths and oscillator strengths (*f*) for **DBC-Si** estimated by TD-DFT calculation [TD-B3LYP-D3/6-31G(d,p)// B3LYP/6-31G(d,p)]

Excited state	Wavelength (nm)	*f*	Transition ($\psi_{xxx} \rightarrow \psi_{yyy}$)					
1	377.71	0.1275	195 → 198	0.15828	196 → 197 (HOMO→LUMO)	0.66980		
2	373.16	0.0094	195 → 197	-0.36578	196 → 198	0.58965		
3	363.25	0.0013	194 → 197	-0.17595	196 → 199	0.67802		
4	336.34	0.1321	195 → 197	0.46872	195 → 198	-0.10438	196 → 198	0.34027
			196 → 200	0.37335				
5	316.65	0.0159	194 → 197	0.34173	195 → 199	0.58542	196 → 199	0.11324

3. General

All the DBC-derivatives reported here were synthesized according to our previous paper.[S1] The electrochemical studies were performed on a BAS ALS842D voltammetry analyzer. Absorption spectra were measured on a JASCO V-770 spectrometer using a quartz cuvette (1 cm square). Emission spectra were measured on a JASCO FP-8500 spectrometer. Concentration for the spectroscopic measurements is as follows, **DBC-H**: 1.03 x 10^{-5} M, **DBC-Me**: 3.97 x 10^{-6} M, **DBC-SMe**: 4.00 x 10^{-6} M, **DBC-Br**: 3.98 x 10^{-6} M, **DBC-S(O)$_2$Me**: 4.00 x 10^{-6} M, and **DBC-Si**: 1.00 x 10^{-5} M. Quantum yield was measured based on the absolute quantum yield method using an integrating sphere (JASCO ILF-835). All calculations were conducted using a Gaussian 16 suite program (G16RevC.01).[S2] In the present study, the optimization for **DBC-H(56°)-1** and **DBC-H(56°)-2** was performed at the B3LYP/6-31G(d,p) level of theory (the calculation for other DBC derivatives were reported in reference S1). Harmonic vibration frequency analysis was conducted with the optimized structures at the same level of theory to verify all stationary points as local minima (with no imaginary frequency). TD-DFT calculations for **DBC-H**, **DBC-Me**, **DBC-SMe**, **DBC-Br**, **DBC-S(O)$_2$Me**, and **DBC-Si** based on B3LYP-D3/6-31G(d,p) were performed using the optimized structures based on B3LYP-D3/6-31G(d,p). See also reference S1 for the DFT calculation of **DBC-H**, **DBC-Me**, **DBC-SMe**, **DBC-Br**, **DBC-S(O)$_2$Me**, and **DBC-Si**.

References

S1. Kamiguchi, S.; Akasaka, R.; Yoshida, N.; Imai, T.; Yamaoka, Y.; Amaya, T.; Iwasawa, T. *Tetrahedron Lett.*, **2022**, *92*, 153664.

S2. M. J. Frisch, G. W. Trucks, H. B. Schlegel, G. E. Scuseria, M. A. Robb, J. R. Cheeseman, G. Scalmani, V. Barone, B. Mennucci, G. A. Petersson, H. Nakatsuji, M. Caricato, X. Li, H. P. Hratchian, A. F. Izmaylov, J. Bloino, G. Zheng, J. L. Sonnenberg, M. Hada, M. Ehara, K. Toyota, R. Fukuda, J. Hasegawa, M. Ishida, T. Nakajima, Y. Honda, O. Kitao, H. Nakai, T. Vreven, J. A. Montgomery, Jr., J. E. Peralta, F. Ogliaro, M. Bearpark, J. J. Heyd, E. Brothers, K. N. Kudin, V. N. Staroverov, T. Keith, R. Kobayashi, J. Normand, K. Raghavachari, A. Rendell, J. C. Burant, S. S. Iyengar, J. Tomasi, M. Cossi, N. Rega, J. M. Millam, M. Klene, J. E. Knox, J. B. Cross, V. Bakken, C. Adamo, J. Jaramillo, R. Gomperts, R. E. Stratmann, O. Yazyev, A. J. Austin, R. Cammi, C. Pomelli, J. W. Ochterski, R. L. Martin, K. Morokuma, V. G. Zakrzewski, G. A. Voth, P. Salvador, J. J. Dannenberg, S. Dapprich, A. D. Daniels, O. Farkas, J. B. Foresman, J. V. Ortiz, J. Cioslowski, and D. J. Fox, Gaussian, Inc., Wallingford CT, 2013.

4. Experimental Procedure

Electrochemical experiments: The CVs and SWVs were measured in CH_2Cl_2 containing 5.0 x 10^{-2} M NBu_4BF_4 as a supporting electrolyte under argon at room temperature with a three-electrode system consisting of a platinum working electrode (BAS), a platinum auxiliary electrode (BAS), and an Ag/AgCl (0.01 M) reference electrode (BAS) at 100 mV/s for CV and 40 mV/s for SWV scan rates. Concentration of DBC derivatives is as follows, **DBC-H**: 1.00 x 10^{-3} M, **DBC-Me**: 1.00 x 10^{-3} M, **DBC-SMe**: 0.99 x 10^{-3} M, **DBC-Br**: 0.97 x 10^{-3} M, **DBC-S(O)$_2$Me**: 1.00 x 10^{-3} M, and **DBC-Si**: 1.00 x 10^{-3} M. Redox potentials are given vs Fc/Fc^+.

5. Cartesian Coordinates of Optimized Structures

DBC-H(56°)-1, optimized at the B3LYP/6-31G(d,p) level of theory.

	Coordinates (Angstroms)		
Atomic Type	X	Y	Z
8	-4.645495	-2.786450	2.335174
8	4.485653	-3.043348	-2.415000
8	4.645495	2.786450	2.335174
8	-4.485653	3.043348	-2.415000
6	1.128883	-2.675016	-1.019320
6	2.489014	0.573785	0.305220
6	1.281584	2.604148	0.940113
6	1.281584	1.335774	0.326084
6	2.450967	-0.715770	-0.395075
6	1.200836	-1.408723	-0.408823
6	0.020441	0.695464	-0.040961
6	-0.020441	-0.695464	-0.040961
6	2.223159	-3.278581	-1.629049
6	3.539014	-1.256248	-1.130522
6	3.562065	2.336408	1.591056
6	2.411041	3.144066	1.542292
6	3.418759	-2.540683	-1.681392
6	-3.609720	-1.049532	1.039181

6	-2.450967	0.715770	-0.395075
6	3.609720	1.049532	1.039181
6	-3.562065	-2.336408	1.591056
6	-1.281584	-1.335774	0.326084
6	-2.489014	-0.573785	0.305220
6	-1.281584	-2.604148	0.940113
6	-3.418759	2.540683	-1.681392
6	-1.128883	2.675016	-1.019320
6	-1.200836	1.408723	-0.408823
6	2.098524	-4.638888	-2.304502
6	-3.539014	1.256248	-1.130522
6	-2.223159	3.278581	-1.629049
6	2.001023	4.298759	3.743524
6	2.384602	4.488021	2.261636
6	-2.411041	-3.144066	1.542292
6	1.089604	-4.605916	-3.468243
6	-2.384602	-4.488021	2.261636
6	-2.098524	4.638888	-2.304502
6	-5.704455	-3.353651	1.566543
6	5.469559	-3.724286	-1.639834
6	1.742646	-5.747537	-1.296680
6	5.704455	3.353651	1.566543
6	1.479183	5.529007	1.586050
6	-1.742646	5.747537	-1.296680
6	-5.469559	3.724286	-1.639834
6	-1.089604	4.605916	-3.468243
6	-1.479183	-5.529007	1.586050
6	-2.001023	-4.298759	3.743524
1	0.166290	-3.167725	-1.080526
1	0.347499	3.146600	1.004562
1	-0.347499	-3.146600	1.004562
1	-0.166290	3.167725	-1.080526
1	3.076840	-4.872630	-2.733627
1	2.023443	5.256654	4.275484
1	2.695539	3.615582	4.239561

1	0.990799	3.884363	3.834046
1	3.406595	4.880699	2.246759
1	1.059573	-5.575898	-3.976856
1	1.362418	-3.843926	-4.204322
1	0.076059	-4.384356	-3.116737
1	-3.406595	-4.880699	2.246759
1	-3.076840	4.872630	-2.733627
1	-6.115871	-2.637102	0.845435
1	-6.486467	-3.638684	2.273850
1	-5.369076	-4.245094	1.020582
1	5.054361	-4.624033	-1.167096
1	5.889133	-3.080328	-0.856892
1	6.265138	-4.015979	-2.329164
1	0.757588	-5.579103	-0.848044
1	2.472940	-5.797698	-0.482406
1	1.718876	-6.724899	-1.791284
1	6.486467	3.638684	2.273850
1	5.369076	4.245094	1.020582
1	6.115871	2.637102	0.845435
1	0.420533	5.255089	1.646929
1	1.733904	5.657672	0.528979
1	1.588500	6.499734	2.080905
1	-2.472940	5.797698	-0.482406
1	-1.718876	6.724899	-1.791284
1	-0.757588	5.579103	-0.848044
1	-5.889133	3.080328	-0.856892
1	-6.265138	4.015979	-2.329164
1	-5.054361	4.624033	-1.167096
1	-0.076059	4.384356	-3.116737
1	-1.059573	5.575898	-3.976856
1	-1.362418	3.843926	-4.204322
1	-1.733904	-5.657672	0.528979
1	-1.588500	-6.499734	2.080905
1	-0.420533	-5.255089	1.646929
1	-0.990799	-3.884363	3.834046

1	-2.023443	-5.256654	4.275484
1	-2.695539	-3.615582	4.239561
1	-4.472349	0.713109	-1.230740
1	-4.509979	-0.452438	1.135441
1	4.509979	0.452438	1.135441
1	4.472349	-0.713109	-1.230740

DBC-H(56°)-2, optimized at the B3LYP/6-31G(d,p) level of theory.

	Cooedinates (Angstroms)		
Atomic Type	X	Y	Z
8	-4.649443	-2.83332	2.282223
8	4.419558	-3.189585	-2.357886
8	4.649443	2.83332	2.282223
8	-4.419558	3.189585	-2.357886
6	1.101155	-2.686549	-1.017934
6	2.494815	0.54801	0.306606
6	1.308451	2.590753	0.941499
6	1.295332	1.322446	0.32747
6	2.443432	-0.741083	-0.393689
6	1.186201	-1.421069	-0.407437
6	0.027633	0.695215	-0.039575
6	-0.027633	-0.695215	-0.039575
6	2.189129	-3.3014	-1.627663
6	3.525831	-1.292785	-1.129136
6	3.58604	2.29944	1.592442
6	2.443432	3.11896	1.543678
6	3.392297	-2.575908	-1.680006
6	-3.620383	-1.01214	1.040567
6	-2.443432	0.741083	-0.393689
6	3.620383	1.01214	1.040567
6	-3.58604	-2.29944	1.592442
6	-1.295332	-1.322446	0.32747
6	-2.494815	-0.54801	0.306606
6	-1.308451	-2.590753	0.941499

6	-3.392297	2.575908	-1.680006
6	-1.101155	2.686549	-1.017934
6	-1.186201	1.421069	-0.407437
6	2.050431	-4.660346	-2.303116
6	-3.525831	1.292785	-1.129136
6	-2.189129	3.3014	-1.627663
6	2.045379	4.277832	3.74491
6	2.430895	4.463116	2.263022
6	-2.443432	-3.11896	1.543678
6	1.041905	-4.61694	-3.466857
6	-2.430895	-4.463116	2.263022
6	-2.050431	4.660346	-2.303116
6	-5.842263	-2.077476	2.386711
6	5.656573	-2.512675	-2.481019
6	1.683105	-5.765254	-1.295294
6	5.842263	2.077476	2.386711
6	1.536292	5.513412	1.587436
6	-1.683105	5.765254	-1.295294
6	-5.656573	2.512675	-2.481019
6	-1.041905	4.61694	-3.466857
6	-1.536292	-5.513412	1.587436
6	-2.045379	-4.277832	3.74491
1	0.133517	-3.169275	-1.07914
1	0.380026	3.142838	1.005948
1	-0.380026	-3.142838	1.005948
1	-0.133517	3.169275	-1.07914
1	3.026277	-4.904194	-2.732241
1	2.077706	5.235443	4.27687
1	2.732791	3.587508	4.240947
1	1.030923	3.873907	3.835432
1	3.457386	4.843585	2.241255
1	1.001844	-5.586559	-3.97547
1	1.322587	-3.857812	-4.202936
1	0.030707	-4.384908	-3.115351
1	-3.457386	-4.843585	2.241255

1	-3.026277	4.904194	-2.732241
1	-5.682232	-1.137551	2.930773
1	-6.545238	-2.698109	2.944873
1	-6.266862	-1.84955	1.400135
1	6.105266	-2.302289	-1.501112
1	5.552111	-1.570039	-3.033971
1	6.312973	-3.183006	-3.038505
1	0.699842	-5.586641	-0.846658
1	2.412841	-5.822966	-0.48102
1	1.649227	-6.742318	-1.789898
1	6.545238	2.698109	2.944873
1	6.266862	1.84955	1.400135
1	5.682232	1.137551	2.930773
1	0.474866	5.250458	1.648315
1	1.79233	5.639435	0.530365
1	1.655643	6.482956	2.082291
1	-2.412841	5.822966	-0.48102
1	-1.649227	6.742318	-1.789898
1	-0.699842	5.586641	-0.846658
1	-5.552111	1.570039	-3.033971
1	-6.312973	3.183006	-3.038505
1	-6.105266	2.302289	-1.501112
1	-0.030707	4.384908	-3.115351
1	-1.001844	5.586559	-3.97547
1	-1.322587	3.857812	-4.202936
1	-1.79233	-5.639435	0.530365
1	-1.655643	-6.482956	2.082291
1	-0.474866	-5.250458	1.648315
1	-1.030923	-3.873907	3.835432
1	-2.077706	-5.235443	4.27687
1	-2.732791	-3.587508	4.240947
1	-4.455747	0.743438	-1.194487
1	-4.504968	-0.392028	1.103239
1	4.504968	0.392028	1.103239
1	4.455747	-0.743438	-1.194487
